# Roles of Dopamine 2 Receptor Isoforms and G Proteins in Ethanol Regulated Prolactin Synthesis and Lactotropic Cell Proliferation

**DOI:** 10.1371/journal.pone.0045593

**Published:** 2012-09-18

**Authors:** Amitabha Sengupta, Dipak K. Sarkar

**Affiliations:** Endocrine Program, Department of Animal Sciences, Rutgers University, New Brunswick, New Jersey, United States of America; University of Cordoba, Spain

## Abstract

Alcohol consumption has been shown to increase prolactin (PRL) production and cell proliferation of pituitary lactotropes. It also causes a reduction in the lactotrope's response to dopaminergic agents and a differential expression of dopamine 2 receptor short (D2S) and long (D2L) isoforms in the pituitary. However, the role of each of these D2 receptor isoforms and its coupled G protein in mediation of ethanol actions on lactotropes is not known. We have addressed this issue by comparing ethanol effects on the level of PRL production gene transcription rate cellular protein, G proteins and cell proliferation in enriched lactotropes and lactotrope-derived PR1 cells containing various D2 receptor isoforms. Additionally, we determined the effects of G protein blockade on ethanol-induced PRL production and cell proliferation in these cells. We show here that the D2 receptor, primarily the D2S isoform, is critically involved in the regulation of ethanol actions on PRL production and cell proliferation in lactotropes. We also present data to elucidate that the presence of the pertussis toxin (PTX)-sensitive D2S receptor is critical to mediate the ethanol stimulatory action on Gs and the ethanol's inhibitory action on Gi3 protein in lactotropes. Additionally, we provide evidence for the existence of an inhibitory action of Gi3 on Gs that is under the control of the D2S receptor and is inhibited by ethanol. These results suggest that ethanol via the inhibitory action on D2S receptor activity suppresses Gi3 repression of Gs expression resulting in stimulation of PRL synthesis and cell proliferation in lactotropes.

## Introduction

Chronic drinking of alcohol has been shown to elevate blood levels of PRL resulting in hyperprolactinemia and various reproductive dysfunctions in both humans and animals [1]–[7]. Using Fischer-344 female rats as an animal model, we have previously shown that ethanol increases and potentiates estradiol stimulatory action on plasma levels of PRL, pituitary PRL content and lactotropic cell proliferation [8]. Furthermore, ethanol stimulates both basal and estradiol-induced PRL secretion and PRL production, as well as, lactotropic cell proliferation in primary cultures of rat pituitary cells [9]. However, how ethanol increases PRL production and lactotropic cell proliferation are not well understood.

Dopamine secreted from the hypothalamus into hypophysial portal vessels is the major inhibitor of PRL production and secretion [10], [11]. Dopamine's inhibitory action of PRL is mediated by the dopamine D2 receptor that belongs to the pertussis toxin (PTX)-sensitive Gi/Go protein coupled receptor family [12]. Recent studies have provided evidence for an inhibitory effect of alcohol on dopaminergic neurotransmission [13]. Dopamine D2 receptors in the brain are decreased in alcoholic patients [14]–[17]. Ethanol also decreases dopaminergic agent response in lactotropes of the pituitary by increasing splicing of D2L receptor mRNA to more D2L variant and less D2S variant (24). Dopamine D2 receptor activation in lactotropes leads to the increased signaling of PTX-sensitive G proteins, Gi/Go, the inhibition of adenylyl cyclase, and the reduction in the intercellular level of cAMP [18], [19]. Abnormalities in dopamine D2 receptors and dopamine transporter function result in hyperplasia of lactotropes [20]–[23]. The D2 receptor is a 7-transmembrane segment protein with a long third intracellular loop and a short intracellular C-terminus. The sixth exon of the D2 receptor gene is often excluded in the mature transcript, resulting in a short (29 amino acids shorter) isoform (D2S). Ethanol strongly favors the expression of the long isoform (D2L) mRNA over the short isoform D2S in the pituitary both in vivo and in vitro [24]. It is not known how ethanol-induced D2 receptor splicing affects the expression of G proteins and changes PRL synthesis and cell proliferation in the lactotrope. This study was conducted to determine the role of D2S and D2L receptor in mediation of ethanol effect on PRL production and lactotropic.

**Figure 1:**
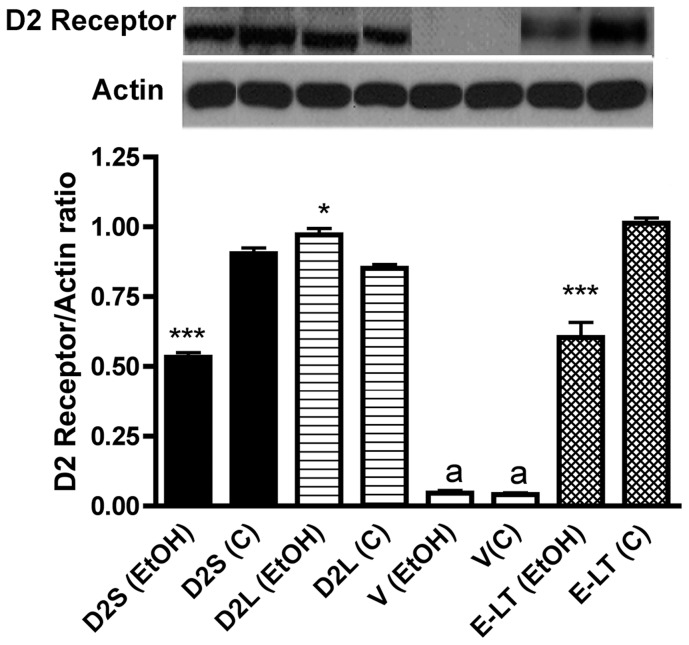
Changes in D2 receptor protein levels in lactotropic cells expressing D2S receptor isoform (D2S), D2L receptor isoform (D2L) or undetectable D2 receptors (V) and in enriched lactotropes (E–LT) following treatment with ethanol for a period of 24 h. The level of D2 receptor protein in all cells was measured by using 25 mg of cellular proteins of each cell in Western blots. Actin was used as control housekeeping protein. In each column of the figure, representative blots were shown on the top and mean ± SEM values of D2 receptor protein and actin ratios were presented as histograms on the bottom. N = 4. *,***, *P*<0.05, *P*<0.001, respectively as compared to control (C)-treated group within a cell type. ^a^, *P*<0.05, significantly different from the rest of the groups.

### Ethic Statement

Animal surgery and care were performed in accordance with institutional guidelines and complied with the National Institutes of Health policy. All experimental procedures and animal treatment protocols were approved by Rutgers Animal Care and Facilities Committee and complied with National Institutes of Health policies.

**Figure 2:**
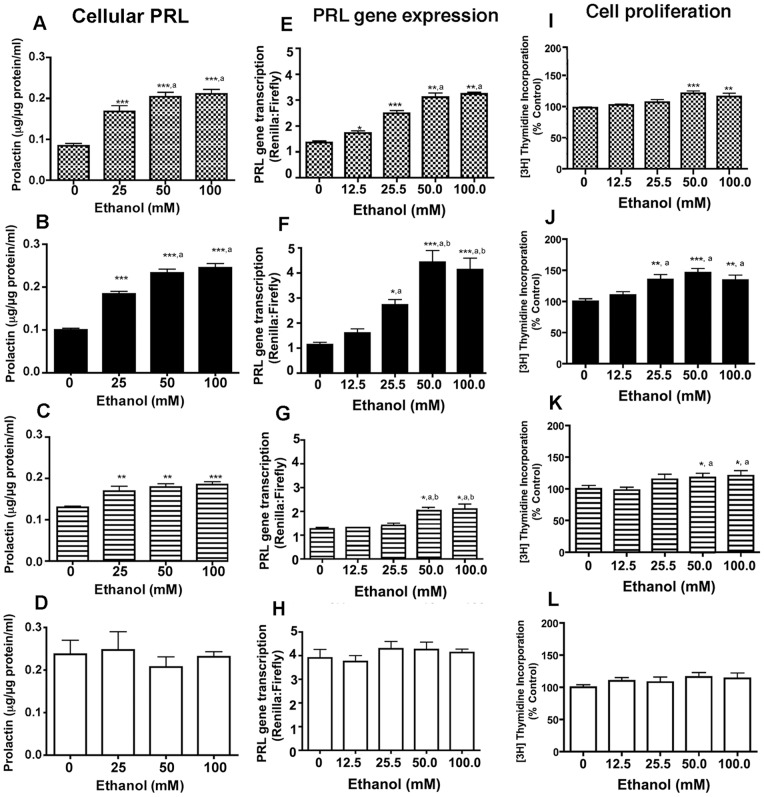
Effect of ethanol on cellular levels of PRL (A–D), PRL gene transcription (E–H) and cell proliferation as measured by [^3^H]thymidine incorporation (I–L) in enriched lactotropes (E-LT; A, E, I) or lactotropic cells expressing D2S receptor isoform (D2S; B, F, J), D2L receptor isoform (D2L; C, G, K) or undetectable D2 receptors (V; D, H, L) cells following treatment with different doses of ethanol for a period of 24 h. PRL gene transcription was measured by luciferase reporter activity. Cellular levels of PRL weremeasured by ELISA. Lactotropic cell proliferation was determined by measuring the [^3^H]thymidine incorporation into cells. Data are mean ± SEM values from 3–4 independent experiments. *, **,***, *P*<0.05, *P*<0.01, *P*<0.001, respectively as compared to “0”- dose- (vehicle)-treated group. ^a^, *P*<0.05, significantly different from the “12.5 mM”- dose- treated group. ^b^ *P*<0.05, significantly different from the “25 mM”- dose- treated group.

## Materials and Methods

### Primary cultures of enriched lactotropes

In limited experiments, enriched lactotropes (E-LT) were used. Anterior pituitaries from female Fisher 344 rats were used to prepare E-LT (about 75–80% lactotropes) using the percol gradient method [25] and maintained in primary cultures. Animal surgery and care were performed in accordance with institutional guidelines and complied with the National Institutes of Health policy. The animal protocol used was approved by the Rutgers Animal Care and Facilities Committee. Cells were maintained at 37°C in 7.5% CO_2_ for 72 h in phenol red-free Dulbecco's Modified Eagle's Medium (DMEM; Sigma, St. Louis, MO) containing 10% fetal bovine serum (FBS), then for 24 h in serum-free DMEM containing serum supplement (SS) consisting of human transferrin (100 µM), insulin (5 µM), putrescine (1 µM) and sodium selenite (30 µM) prior to treatment with the tested agent. We used 0.25 million cells/well for PRL production (gene transcription and cellular protein content) experiments and 0.5 million cells/well for cell proliferation studies. Cultures were treated with various doses of ethanol for a period of 24 h.

**Figure 3:**
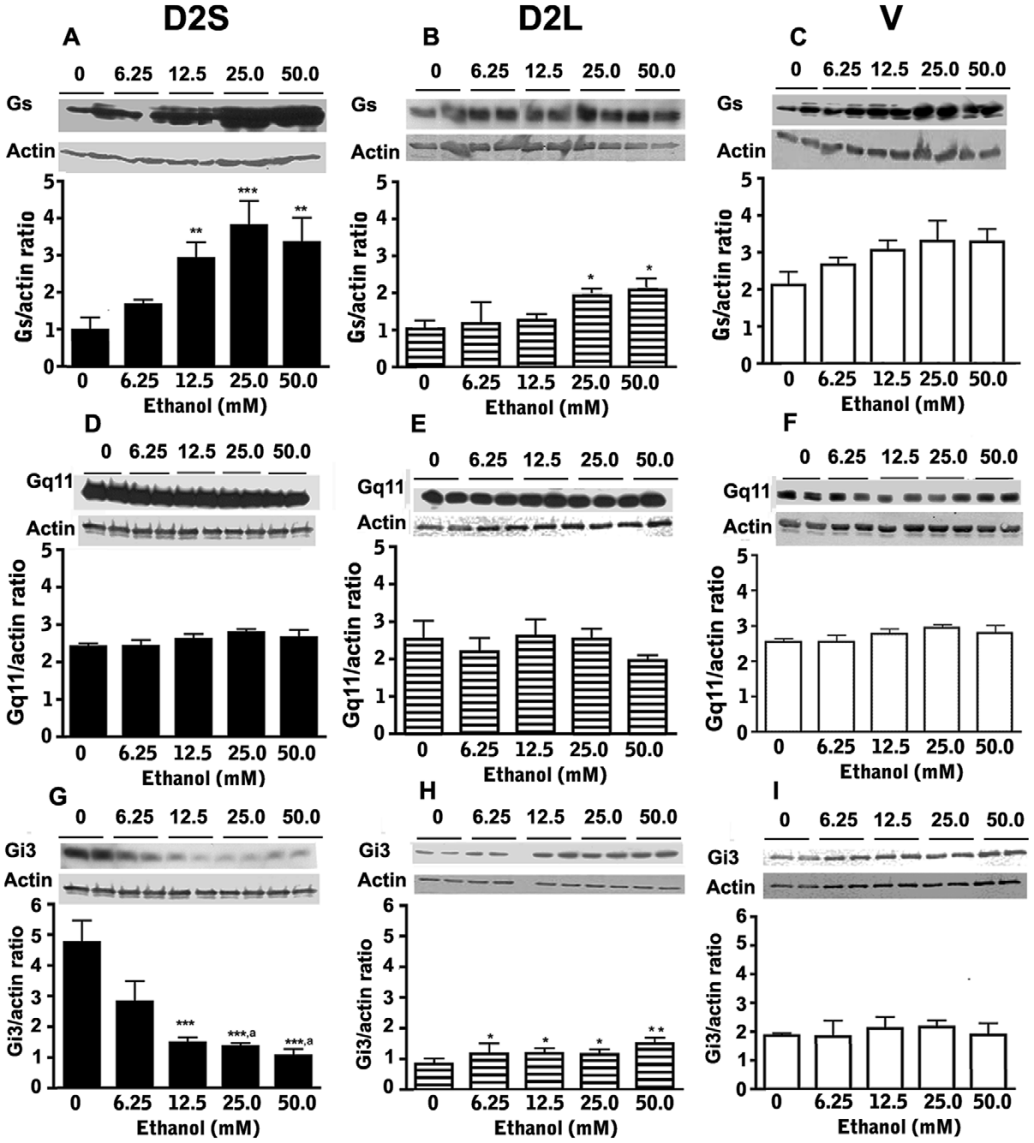
Effects of ethanol on cellular levels of G proteins (Gs, Gq11 and Gi3) in D2S (A, D, G), D2L (B, E, H) and V (C, F, I) cells following treatment with different doses of ethanol (0–50 mM) for a period of 24 h. Quantitation of G protein levels was done using Western Blot and actin was used as a control housekeeping protein. Data were presented in gel blots as well as the ratio of the protein/actin values as histograms. Data are mean ± SEM values of 4 cultures. *, **,***, *P*<0.05, *P*<0.01, *P*<0.001, respectively as compared to “0”- dose- (vehicle)-treated group. ^a^, *P*<0.05, significantly different from the “12.5 mM”- dose- treated group.

### PR1 cells expressing various amount of dopamine D2 receptors

We used a well-characterized PRL secreting PR1 cell line [26]–[28]. Previously, using these cells we have made several stable transfectants expressing undetectable amounts of dopamine D2 receptors (V cells), containing the recombinant D2L receptor (D2L cell) or D2S receptors (D2S cell). We have verified the expression patterns of the D2 receptors in these cell lines [29], [30]. Transfectants of PR1 cells were maintained in a 1∶1 mixture of DMEM and Ham's F-12 medium (DMEM-F-12; Sigma) containing 10% FBS and 800 µg/ml G-418 sulfate (Promega, Madison, WI). We used 0.25 million cells/well in a 12 wells plate for PRL production experiments and 0.5 million cells/well in a 6 wells plate for cell proliferation studies. Prior to the treatment with a tested agent, cells were maintained for 24 h in serum-free DMEM/F-12 with SS. Cells were treated with various doses of ethanol for a period of 24 h in order to determine the dose-response effect of the drug on PRL and G proteins levels and cell proliferation. For determination of the role of dopamine receptors and G proteins in PRL production and cell proliferation, cells were treated with 50 mM of ethanol in the presence or absence of dopamine (DA; 5 µM) and pertussis toxin (PTX; 100 ng/ml) alone or together for a period of 24 h. Cells were lysed and levels of proteins were detected by Western blot or used for PRL gene transfection assay. Cell proliferation was determined by [3H]-thymidine incorporation assay.

**Figure 4:**
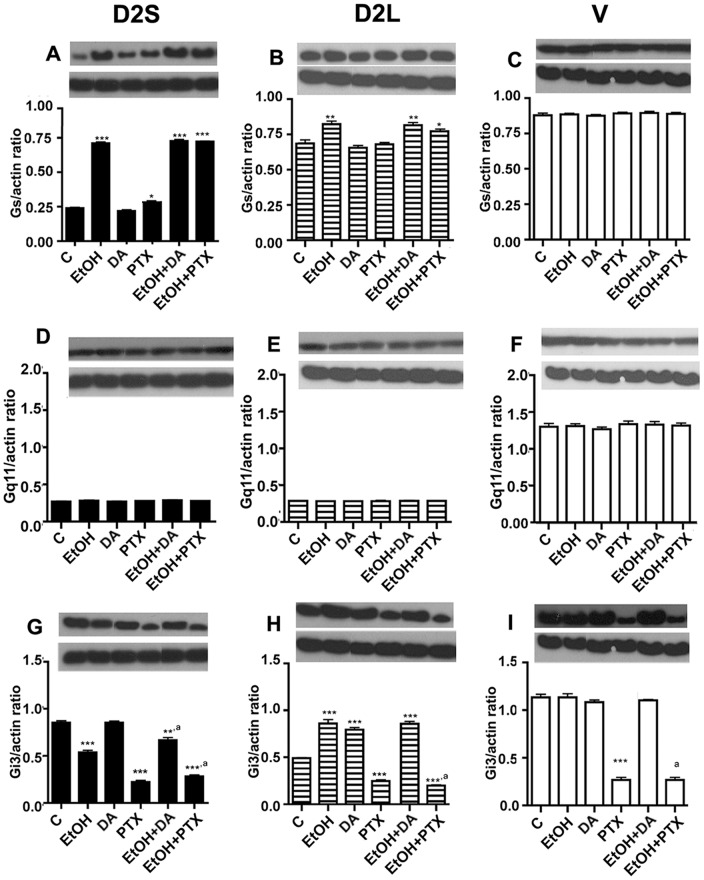
Effects of dopamine and pertussis toxin on ethanol modulated G proteins expression in D2S (A, D, G), D2L (B, E, H) and V (C, F, I) cells. Cells were treated with ethanol (EtOH; 50 mM) alone or with dopamine (DA; 5 µM) or PTX (100 ng/ml) for a period of 24 h. Quantitation of G protein levels was done using Western Blot and actin was used as a control housekeeping protein. Data were presented in gel blots as well as the ratio of the protein/actin values as histograms. Data are mean ± SEM values of 4 cultures. *, **,***, *P*<0.05, *P*<0.01, *P*<0.001, respectively as compared to the control (C) group. ^a^, *P*<0.05, significantly different from PTX alone-treated group.

### Western Blot

Cellular levels of G proteins and PRL were determined by Western blots using the ECL All of the primary and secondary antibodies used in this study were previously characterized [31]. The primary antibodies used were rabbit anti rPRL (1∶30,000), D2 receptor antibody (1∶1000), Gs antibody (1∶1000), rabbit anti Gq11 antibody (1∶1000), rabbit anti Gi3 antibody (1∶1000), and mouse anti-actin antibody (1∶5000). All antibodies were purchased from Santa Cruz Biotechnology (Santa Cruz, CA), except for anti rat PRL, which was obtained from the National Institute of Diabetes and Digestive and Kidney Diseases (NIDDK; PRL-S9) and anti Gi3 and actin antibody which were purchased from Upstate Biotechnology (Cleveland, Ohio) and EMD Chemical (Gibbstown, NJ), respectively. For quantification of protein levels, the same amount of total cellular protein for all the samples was used in the assay. The band intensities of proteins were determined using Scion Image software and normalized to the corresponding actin band intensities. The ethanol treatment protocol used did not affect the level of cellular actin. Hence, G proteins and PRL levels were normalized with actin levels, and the ratios of G protein/actin and PRL/actin were determined and presented in the text and figures.

**Figure 5:**
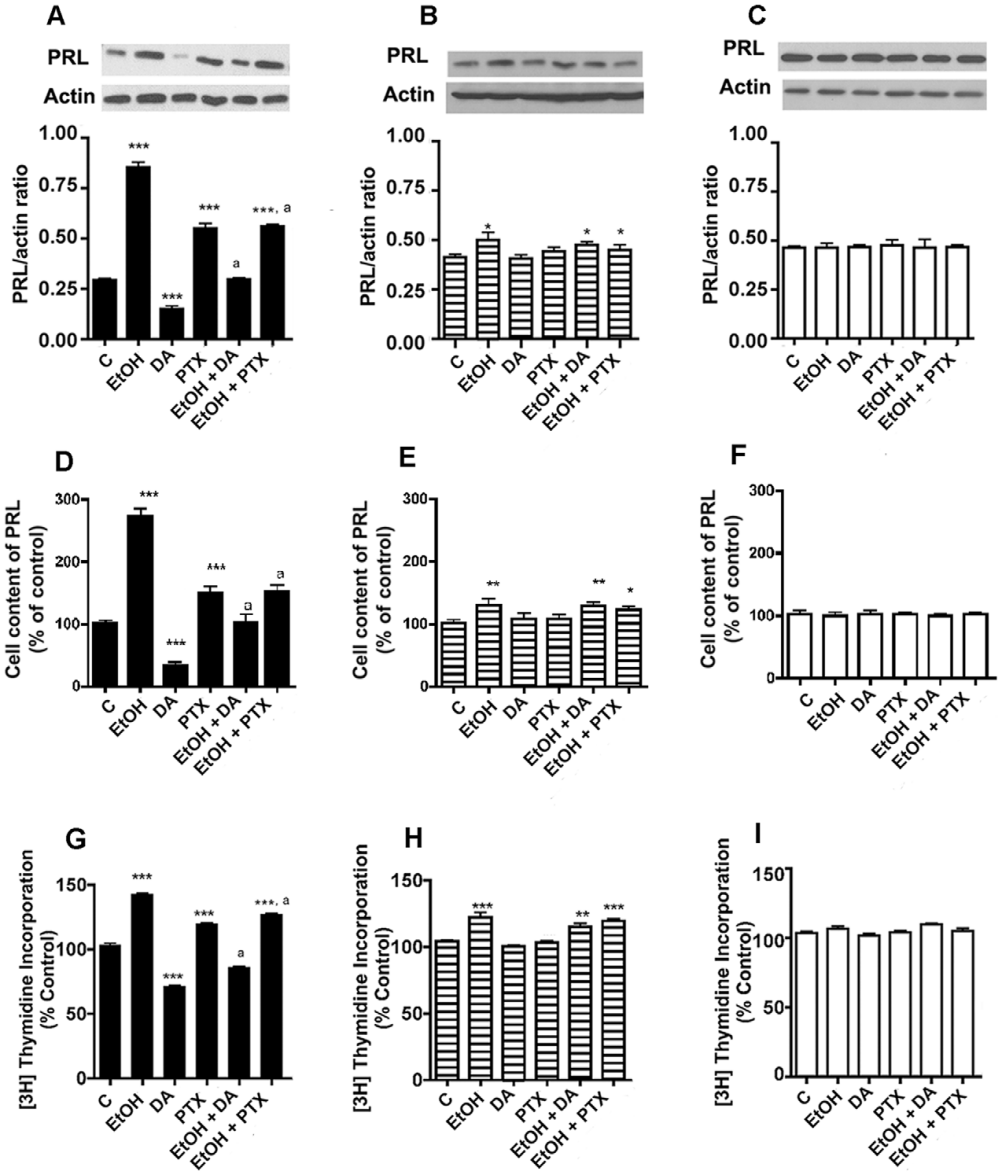
Effects of dopamine and pertussis toxin on ethanol modulated cellular levels of PRL and cell proliferation in D2S (A, D, G), D2L (B, E, H) and V cells (C, F, I). Cells were treated with the dopamine (DA) or pertussis toxin (PTX) as described in the legend of Fig. 4. Cellular levels of PRL were determined by Western blots (A–C) or by ELISA (D–F). Cell proliferation studies were conducted by measuring the [^3^H]thymidine incorporation into cells (G–I). N = 4–9. *, **,***, *P*<0.05, *P*<0.01, *P*<0.001, respectively as compared to the vehicle-treated group (C). ^a^, *P*<0.05, significantly different from the EtOH only treatment.

### PRL gene transcription

PRL gene transcription was measured using luciferase reporter activity and using luciferase reporter construct containing approximately 2.5 kilobase pairs of the 5′-flanking sequence and the promoter from the rat PRL gene [32] provided by Dr. Arthur Gutierrez-Hartmann from UCHSC. Briefly, the cells were transfected by a superfect reagent (QIAGEN, Valencia, CA) with a plasmid containing the luciferase structural gene fused to 2.5 kb of the 5′- flanking region of the rat PRL gene following manufacturer protocols. After transfection, cells were incubated with the serum-free media containing serum supplement for 24 h prior to treatment with various doses of ethanol or vehicle control for 24 hours. The luciferase activity for each culture plate was determined using Dual-glo luciferase assay system (Promega, Madison, WI).

**Figure 6:**
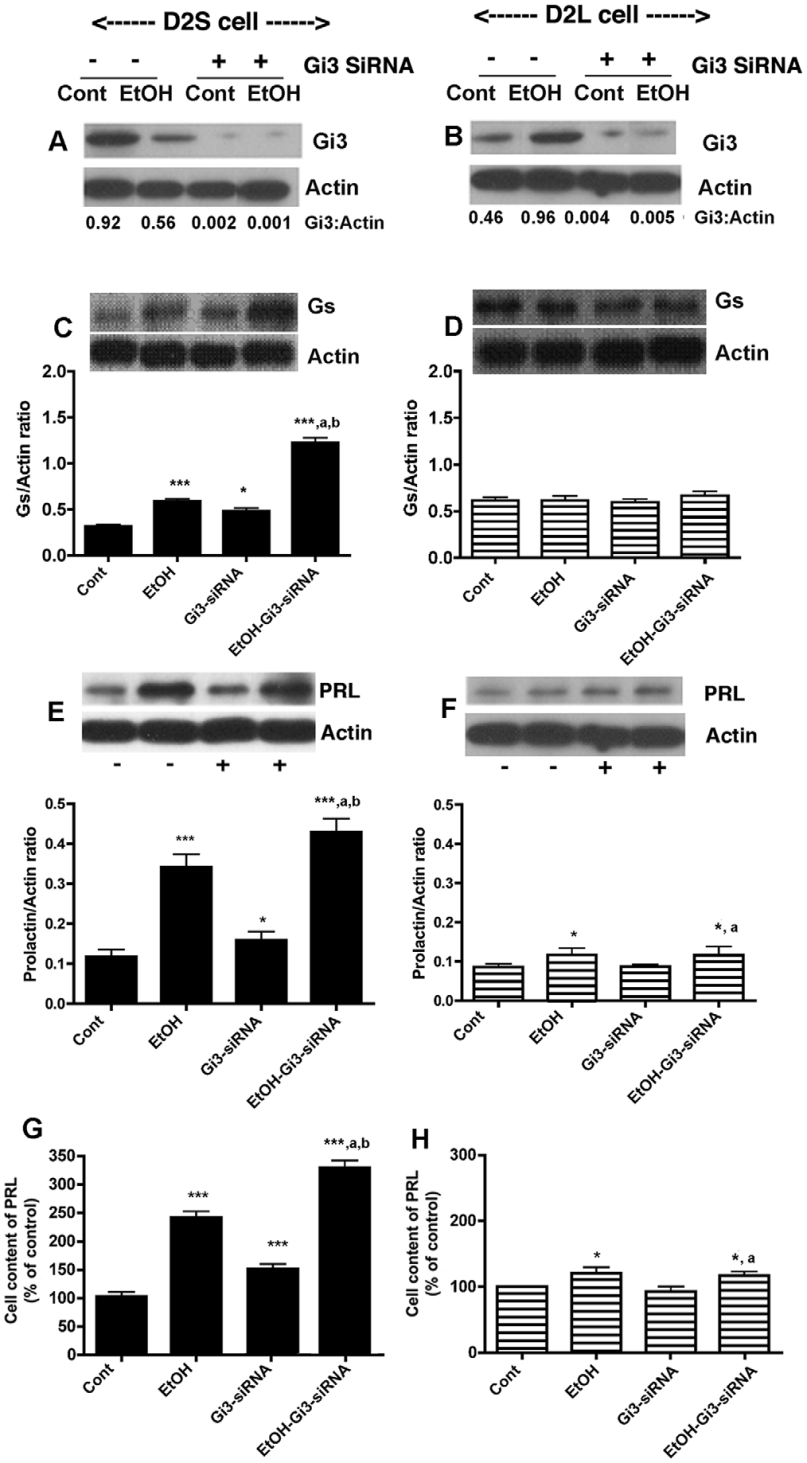
Effect of knock-down of Gi3 proteins by siRNA on ethanol modulated cellular levels of G proteins and PRL in D2S (A, C, E, G) and D2L cells (B, D, F, H). Cells treated with control or 50 mM ethanol (EtOH) were co-incubated with or without siRNA for Gi3 for 24 h. Cellular levels of Gi3 (A, B), Gs (C, D) and PRL (E–F) were determined by Western blots and Actin as a control housekeeping protein. Cellular levels of PRL were verified by ELISA (G, H). N = 4–6. ***, *P*<0.001, *, *P*<0.05, significantly different from the vehicle-treated group (Cont). ^a^, *P*<0.001, significantly different from siRNA only treatment. ^b^, *P*<0.001, significantly different from ethanol only treatment.

### Cell proliferation response

Cell proliferation was determined by the [3H]-thymidine incorporation methods as described by us previously [29], [30]. Each experiment was conducted in duplicate and repeated 3 times. In some experiments, because samples were run in multiple assays, the dpm values of each treatment varied between assays and therefore data were calculated and presented as a % of control.

**Figure 7:**
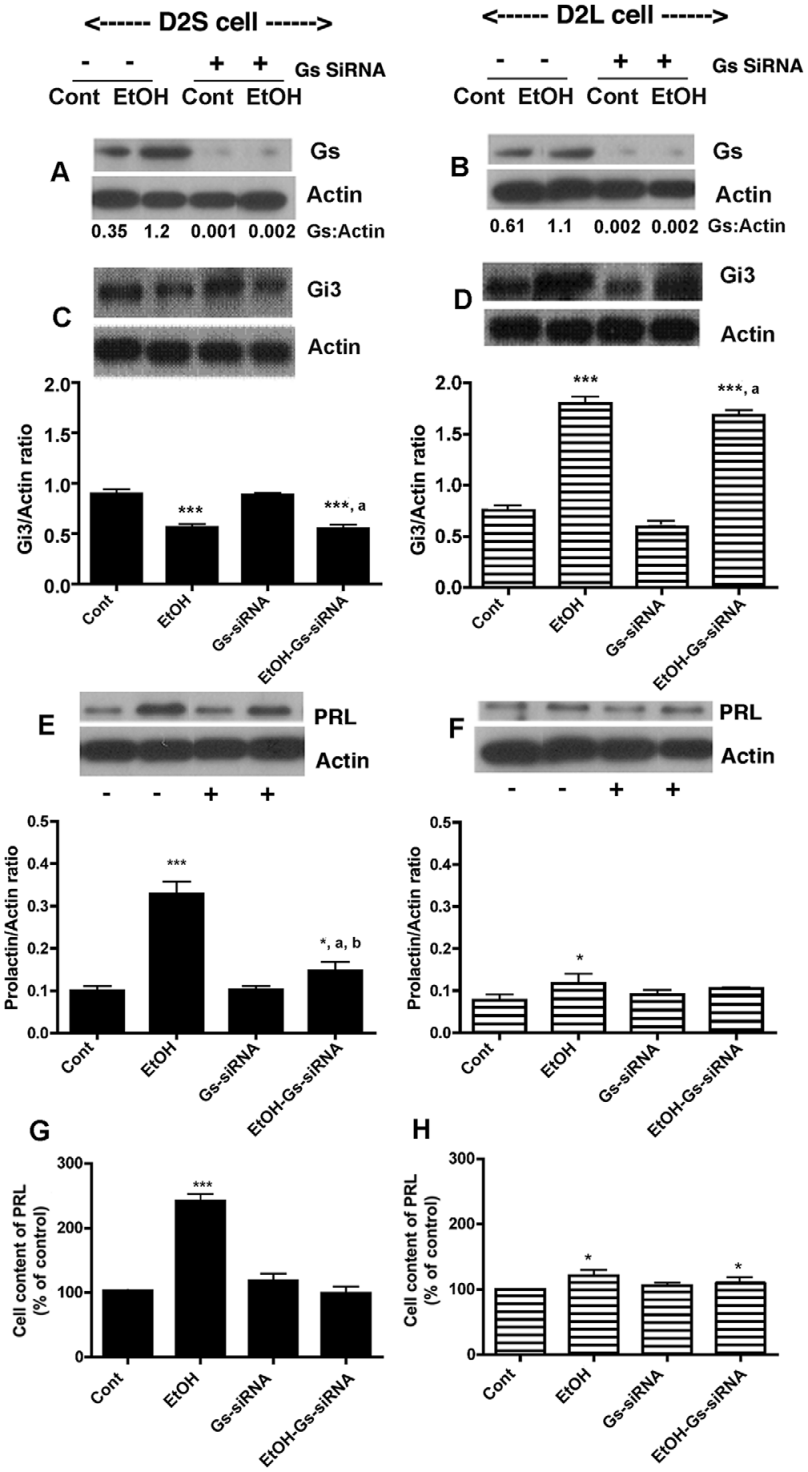
Effect of knock-down of Gs proteins by siRNA on ethanol modulated cellular levels of G proteins and PRL in D2S (A, C, E, G) and D2L cells (B, D, F, H). Cells treated with control or 50 mM ethanol (EtOH) were co-incubated with or without siRNA for Gs for 24 h. Cellular levels of Gs (A, B), Gi3 (C, D) and PRL (E, F) were determined by Western blots and Actin as a control housekeeping protein. Cellular levels of PRL were verified by ELISA (G, H). N = 4–6. ***, *P*<0.001, *, *P*<0.05, significantly different from the vehicle-treated group (Cont). ^a^, *P*<0.001, significantly different from the siRNA only treatment. ^b^, *P*<0.001, significantly different from ethanol only treatment.

### Knock-down of Gi3 and Gs proteins with siRNA

Rat siRNA for Gi3 (Rn_Gnai3_5_HP siRNA; gene accession number NM_013106) and Gs (Rn_Gnas_HP siRNA; gene accession number NM_001024823) were bought from QIAGEN (Valencia, CA) as lyophilized powder. Each of them was 74 mg/tube (5 nmol/tube). D2L and D2S cells were transfected with 37.5 ng/1.5 μl of each siRNA and 3 μl/ml HiPerfect Reagent (QIAGEN) in a final volume of 100 μl serum free culture medium with SS and incubated for 10 minutes at room temperature for complex formation. The transfection mix was then added onto cells with or without 50 mM ethanol and incubated at 37°C and 7.5% CO2 for 24 h and used for proteins measurement by Western blot.

**Figure 8:**
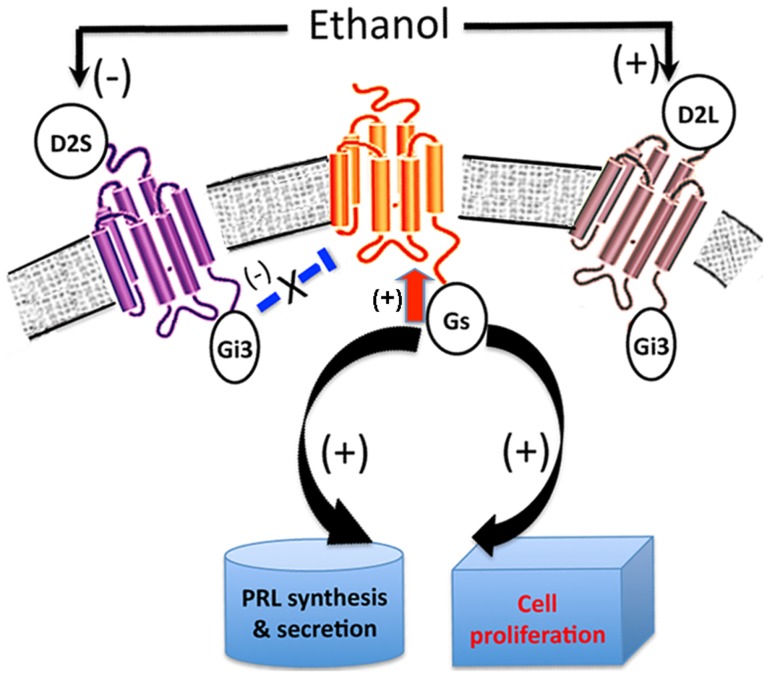
A diagram summarizing the postulated role of D2 receptor signaling in mediation of ethanol action on PRL production and lactotropic cell proliferation. It is hypothesized that ethanol action on PRL production and cell proliferation possibly involves the downregulation of the D2S-coupled-PTX-sensitive Gi3 protein that activates Gs protein-mediated signaling to stimulate PRL production and cell proliferation in lactotropes. Furthermore, by increasing the production of D2L, ethanol may decouple the D2 receptor influence on Gi3 and Gs interaction and thereby reduce the dopaminergic inhibitory control over PRL production and cell proliferation.

### Measurement of PRL levels

Prolactin (PRL) levels in cellular extracts of some samples were verified by ELISA assay (Alpco Immunoassays, NH, USA). 10 μg/ml of cellular extract of the culture was added to the microplate coated wiyh a monoclonal anti-rat PRL antibody. A standard curve between 5 and 80 ng/ml of PRL was utilized to compare the content of PRL in the experimental samples.

### Statistical analysis

The data shown in the figures and text are means ± SEM. Comparisons between two groups were made using *t*-tests. Data comparisons between multiple treatment groups were made using one-way ANOVA. Student-Newmann-Keuls test was used as a post-hoc test. Graph Pad Prism 4.0 (Graph Pad Software, Inc., San Diego, CA) was used for statistical analyses and graph preparation. A value of *P*<0.05 was considered significant.

## Results

### Effects of ethanol on D2 receptors in cells expressing various D2 receptor isoform variants

Ethanol is known to increase PRL production by reducing D2R levels via differential splicing of the gene in lactotropes [24]. We first characterize whether differential expression of D2L and D2S receptors affects ethanol's ability to alter D2R production in lactotropic cells. We used E-LT cells containing intact D2 receptors, V cells containing no D2 receptors, D2L cells containing only D2 receptor long isoform, and D2S cells containing only D2 receptor short isoform [30]. Measurement of D2 receptor protein levels indicated that E-LT cells, D2L and D2S cells contain similar level of this receptor protein while V cells had no detectable amount of this receptor protein (Fig. 1). Treatment with 50 mM dose of ethanol markedly decreased D2R level in D2S and E-LT cells but moderately increased the D2R in D2L cells. Ethanol treatment did not change the very low level of D2R in V cells. Since D2S cells express only D2S mRNA variant and D2L cells produce only D2L variant, the changes in D2R protein levels possibly reflect the changes in the expression of these gene variants.

### Effect of ethanol on cellular PRL, PRL gene transcription and cell proliferation in enriched lactotropes and lactotropic cells expressing D2S, D2L, or no D2 receptors

We studied whether differential expression of D2L and D2S receptors affects ethanol's ability to alter lactotropic cell functions by comparing the changes in PRL gene transcription rate, cellular level of PRL, and the cell proliferation rate in E-LT, D2S, D2L and V cells. Ethanol increased the level of PRL and the PRL gene transcription rate in E-LT and D2S cells in a concentration-dependent manner (Fig. 2A, B, E, F). In both of these cell types, a dose of 50 mM was effective in inducing the maximum effect. Ethanol also moderately increased PRL levels and PRL gene transcription rates in D2L cells, but was not able to significantly increase in V cells (Fig 2C, D, G, H). Ethanol effect on the cell proliferation response, as measured by [^3^H]-thymidine incorporation in cells, also revealed similar differences in its action on E-LT, D2S, D2L and V cells (Fig. 2E–H). Ethanol was most effective in increasing cell proliferation in D2S cell and E-LT cells, moderately effective in D2L cells, and not effective in V cells (Fig. 2I–L). These data indicate that ethanol actions on PRL production and cell proliferation require the presence of D2 receptors. Additionally, the data show the preference of the D2S receptor over the D2L receptor in ethanol action on PRL production and cell proliferation in lactotropes.

### Effect of ethanol on G proteins expression in lactotropic cells expressing various D2 receptor isoforms

Dopamine D2 receptors are G protein coupled, and the pituitary levels of specific groups of G proteins (Gi3 and Gs) are shown to be highly responsive to the effect of ethanol, whereas Gq11 and other G proteins (Gi1, Gi2) are insensitive or very moderately responsive to ethanol in vivo [31]. Hence, we determined ethanol-induced changes in Gi3, Gs and Gq11 proteins in cell lines expressing various D2 receptor isoforms as well as in enriched lactotropes. As shown in Figure 3A–C, ethanol treatment markedly increased the cellular level of Gs in D2S and moderately increased in D2L cells but produced no effect on cellular levels of Gs in V cells. Figure 3D–F shows that ethanol treatment markedly decreased Gi3 levels in D2S cells but significantly increased Gi3 levels in D2L cells and produced no effect on Gi3 levels in V cells. Ethanol failed to change Gq11 levels in any of these cells (Fig. 3G–1). Determination of ethanol effect, using its maximal effective dose (50 mM), on E-LT revealed that ethanol significantly increased (% of “0”-dose achieved by 50 mM ethanol) Gs levels (124.0±2.1; N = 8), decreased Gi3 levels (47.8±2.2, N = 8), and produced no changes of Gq11 levels (100.1±0.3; N = 8) in E-LT cells. These data also identified the preference of D2S over D2L in mediating ethanol stimulatory action on the cellular level of Gs protein and the inhibitory action on Gi3 protein. Additionally, these data revealed a differential action of ethanol on the cellular level of Gi3 protein under the influence of D2S and D2L receptors.

### Effects of dopamine and pertussis toxin on ethanol modulated G proteins expression in lactotropic cells expressing D2S, D2L or no D2 receptors

Previous studies identified that D2 receptors are positively coupled with PTX-sensitive Gi3 proteins, which inhibit Gs protein expression to reduce the cellular level of cAMP and thereby PRL gene production and cell proliferation in lactotropes [33]. Although D2L and D2S receptors have similar pharmacological and biochemical profiles [34], the data shown in Fig. 2 identify, in the presence of ethanol, the differential effects of D2S and D2L receptors on Gi3 and Gs proteins. Hence, the question arose as to whether ethanol effect on D2 ligands-modulated G proteins depends on the influence of D2S and/or D2L receptors. In order to investigate this, we tested the effect of PTX and dopamine (DA) on the ethanol-induced alteration of Gi and Gs proteins, and as control, on Gq11 protein. As shown in Fig. 4A, in D2S cells, treatment of dopamine produced no effect on the basal or the ethanol-induced level of Gs protein, while PTX moderately increased basal Gs levels. In D2Lcells, treatment of PTX, or dopamine produced no effect on the basal or the ethanol-increased levels of Gs protein in D2L cells (Fig. 4B) or V cells (Fig. 4C). None of the treatment affected Gq11 levels in D2S, D2L or V cells (Fig. 4D–F). DA also produced no effect on the basal level of Gi3 but produced significant blockade of ethanol-induced inhibition of Gi protein in D2S cells (Fig. 4G). PTX reduced the basal level of Gi3 as well as potentiated the ethanol-induced inhibition of Gi protein in D2S cells. In D2L cells, unlike to that observed in D2S cells, DA increased basal levels of Gi3. PTX inhibited ethanol effects on these cells. None of the treatment affected Gi levels in V cells, with the exception of PTX, which decreased Gi3 levels in this cells (Fig. 4I). These data suggest that the D2S receptor is negatively coupled while the D2L receptor is positively coupled to Gi3 protein. Therefore, ethanol suppression of D2S receptors results in a decrease of Gi3 levels while ethanol activation of the D2L receptor causes an increase in cellular levels of the Gi3 protein. The data also identify differential effects of dopaminergic agents on Gi3 protein in D2S and D2L cells.

### Effects of dopamine and pertussis toxin on ethanol modulated cellular PRL and cell proliferation in lactotropic cells expressing D2S, D2L, or no D2 receptors

Ethanol-induced alteration of D2 receptor splicing and their coupling with G proteins effected PRL production and cell proliferation was determined. Similar to lactotropes [29], [35], in D2S cells, DA decreased and PTX increased the basal level of cellular PRL (Fig. 5A, D). In D2S cells, DA and PTX suppressed ethanol-induced PRL production. In D2L cells, DA or PTX produced no significant effect on either basal or ethanol-induced changes in cellular levels of PRL (Fig. 5B, E). In V cells, neither ethanol nor DA alters cellular levels of PRL (Fig. 5C, F).

Like PRL production in D2S cells, DA decreased and PTX increased the basal level of [^3^H]-thymidine incorporation into cells (Fig. 5G). DA and PTX inhibited the ethanol-induced thymidine incorporation into D2S cells. In D2L cells, PTX and DA failed to alter the basal level or ethanol-induced thymidine incorporation into cells (Fig. 5H). In V cells, neither ethanol nor the DA alter cell proliferation, as determined by the thymidine incorporation into cells (Fig. 5I). These data suggest that ethanol action on PRL production and cell proliferation possibly involves downregulation of D2S-coupled-PTX-sensitive G proteins.

### Effects of Gs and Gi3 blocking by siRNA on PRL levels in lactotropic cells expressing D2S, D2L, or no D2 receptors

To further determine the role of D2 receptor isoforms and Gi3 and Gs proteins in ethanol action on PRL production, we also employed an siRNA approach to knock-down the Gi3 or Gs genes and proteins in D2S and D2L cells. Transfection of Gi3 siRNA markedly reduced the cellular level of this protein in D2S (Fig. 6A) and D2L cells (Fig. 6B). Interestingly, Gi3 suppression elevated both basal and ethanol-induced Gs levels but only in D2S cells (Fig. 6C) and not in D2L cells (Fig. 6D), suggesting a possible existence of an inhibitory control of Gi3 over Gs production in the D2S cell. Under Gi3 blockade the basal and ethanol-induced PRL production was significantly increased in D2S cells (Fig. 6E, G), suggesting that the Gi3 blockade increases PRL level. Gi3 blockade did not alter basal or ethanol-stimulated PRL production in D2L cells (Fig. 6F, H). Transfection of Gs siRNA markedly reduced the cellular level of this protein in D2S (Fig. 7A) and D2L cells (Fig. 7B) without affecting the basal and ethanol-induced Gi3 protein levels in these cells (Fig. 7C &D). Gs blockade significantly reduced ethanol effect on PRL in D2S cells (Fig. 7E, G) but not in D2L cells (Fig. 7F, H). These results suggest that there exists an inhibitory interaction between Gi3 on Gs that controls D2S mediated actions on lactotropes. Furthermore, the results suggest the possibility that ethanol, by suppressing Gi3, enhances the Gs regulated PRL production in lactotropes.

## Discussion

The data presented here identify ethanol as a potent stimulator of PRL production and lactotropic cell proliferation. This study also identifies a D2 receptor-dependent mechanism in ethanol action on lactotropes. Additionally, the data provide evidence for the possibility that ethanol suppresses the D2S-regulated coupling of PTX-sensitive Gi3 protein to activate Gs protein-mediated signaling for the stimulation of hormone production and cell proliferation.

The importance of D2 receptor involvement in ethanol actions on PRL production and cell proliferation is evident in the experiment where ethanol actions on lactotropes' function were evaluated in clonal cell lines expressing various levels of D2 receptor isoforms. It was found that V cells, which lacked functional D2 receptors and had high basal levels of PRL, showed a significant reduction in PRL production following D2S and D2L receptor transfection. As described by an extended allosteric ternary complex model of G protein-coupled receptor activation, receptors spontaneously isomerize between active and inactive conformations, so receptors can modulate signaling pathways in the absence of an agonist. In addition, significant constitutive activity of recombinant D2S receptors expressed in mammalian cells has been described previously [36]. Hence, the ligand-independent changes in hormone production were due to constitutively activated D2 receptors in transfected cells. V cells that lacked functional D2 receptors did not respond to ethanol in respect to PRL production. However, ethanol stimulated PRL production in these cells following the D2 receptor transfection, suggesting that the D2 receptor is a prerequisite for ethanol action on PRL.

It is interesting to note that the PRL response to ethanol was higher in D2S cells than in D2L cells. Dopamine D2 receptor exist into two molecularly distinct isoforms, D2L and D2S. Both isoforms, generated by alternative splicing from the same gene, have similar pharmacological and biochemical profiles *in vitro*, despite the presence of an additional 29 amino acids in the D2L isoform [37]–[40]. This additional segment is located in the third intracellular loop of the putative receptor structure, a region involved in the coupling to the G proteins. Ethanol has been shown previously to induce differential expression of D2L and D2S mRNA and therefore alters D2 receptor splicing in pituitary cells. It is interesting to note that estradiol, which stimulates lactotrope's proliferation and PRL production, also favored the production of the long isoform of D2 mRNA over the short one in MMQ cells [41]. Also like that observed for ethanol in this study, estradiol treatment was able to markedly increase PRL production and cell proliferation in cells expressing the D2S receptor, but produced a minimal effect in cell producing D2L receptors [30]. The proportion of messenger RNA corresponding to the D2S but not the D2L was shown to be low in dopamine-resistance compared to dopamine-responsive prolactinomas [40]. Additionally, by using D2L−/− mice it was shown that the function of D2S is not dependent on the formation of a receptor heterodimer with D2L [42]. It has recently been shown that transgenic mice, overexpressing D2S but not D2L, show pituitary hypoplasia [20]. Together these data suggest that the suppression of D2S receptor expression may be a critical step in ethanol-regulated PRL production and lactotropic cell proliferation.

Dopamine D2 receptor activation in lactotropes leads to the alteration of G protein coupling that results in inhibition of adenylyl cyclase, and reduction of intercellular cAMP and PRL production and possibly cell proliferation [18], [19]. The D2S receptor is known to couple to Gi2 to inhibit forskolin-induced cAMP production, while it coupled to Gi3 to inhibit adenylate cyclase activated by a Gs-coupled receptor (PGE1 receptor). D2S-induced increase in [Ca^2++^] is not dependent on any particular Gi/o subtype, but is dependent on mobilization of Gbg subunit [33]. Therefore, the dopamine D2S receptor utilizes different Gi/o protein subunits to regulate a diversity of effector functions within the cell. In this study, we identified ethanol stimulatory action on Gs and inhibitory action on Gi3 in D2S cells but not clearly in D2L cells; in these cells, ethanol moderately stimulated both Gi3 and Gs. In D2S cells ethanol action on Gi3 and PRL production and on cell proliferation were suppressed by dopamine agonist as well as by PTX. Ethanol increased Gs levels in D2S cells that were not inhibited by dopaminergic agents or PTX. Similarly, ethanol's stimulatory action on Gs in D2L cells is minimally affected by dopaminergic agents or PTX. Previous studies identified that D2 receptors are positively coupled with PTX-sensitive Gi3 proteins, which inhibit Gs protein expression to reduce cellular level of cAMP and thereby PRL gene production and cell proliferation in lactotropes [33]. We showed here that a Gi3 blocker was able to stimulate the basal level of PRL while a Gs blocker was able to inhibit ethanol action on PRL production in D2S cells, but not in D2L cells. It is noteworthy that Gi3 suppression by siRNA increased the cellular levels of Gs in D2S cells. In D2S cells ethanol suppressed Gi3 and increased Gs showing a reversal expression of these two proteins under the influence of the drug. On the other hand, in D2L cells ethanol moderately increased both Gs and Gi3, showing a moderate and non-reversal effect on these two G proteins. These data support a concept that there exists a competitive interaction between Gi3 and Gs proteins in lactotropes. Furthermore, the data showing increased Gs levels following Gi3 blockade in ethanol-responsive D2S cells and not in D2L cells identify the critical importance of the Gi3 and Gs inhibitory interaction in the regulation of ethanol action on lactotropic cells.

In summary, the data presented here identify a role of D2S receptor-specific mediation of ethanol action on PRL production and cell proliferation in lactotropes. We propose that during sustained exposure, ethanol cancels the inhibitory effect of dopamine via increasing the ratio of D2L:D2S receptors. This may cause downregulation of D2S-coupled-PTX-sensitive Gi3 protein and resulting in the lowering of Gs protein-mediated signaling to stimulate PRL production and cell proliferation in lactotropes. Furthermore, by increasing the production of D2L, ethanol may decouple the D2 receptor influence on Gi3 and Gs interaction and thereby causing reduction in the dopaminergic inhibitory control over PRL production and cell proliferation (Fig. 8).
